# PRO perspective

**DOI:** 10.1192/j.eurpsy.2021.216

**Published:** 2021-08-13

**Authors:** A. Fiorillo

**Affiliations:** Department Of Psychiatry, University of Campania, Naples, Italy

## Abstract

**Abstract Body:**

The COVID-19 pandemic has had a detrimental impact not only on the ordinary lives of people worldwide, but also on the access to mental health care system. In particular, in the first months of the global health emergency, a drastic reduction in the number of access to healthcare system has been recorded. In the “Phase 1” of the emergency, the fear for the contagion, the strict containment measures and the lack of adequate information regarding the virus have been listed as possible factors contributing to this phenomenon. In the “Phase 2”, mental health care has been completely reorganized in order to compile with requirements for physical distancing and reducing overcrowding. The visits in outpatients’ units have been rescheduled, healthcare professionals have received information regarding the adequate use of protective personal equipment and patients have learnt how to protect themselves. Furthermore, telemental health approaches have been fostered worldwide, although several obstacles still persist such as the lack of adequate training for healthcare professionals for using telemental health instruments, the uncertainties regarding the legal implications of telemental health and the difficulties for older patients to access those systems. During this critical period, mental healthcare systems have been proven to be resilient. The pandemic has speed up the process of transformation of mental health care system and we are learning how to further improve the system in order to sustain these changes in the long-term.

**Disclosure:**

No significant relationships.

